# Differential Sensitivity of Epithelial Cells to Extracellular Matrix in Polarity Establishment

**DOI:** 10.1371/journal.pone.0112922

**Published:** 2014-11-13

**Authors:** Shigenobu Yonemura

**Affiliations:** 1 Electron Microscope Laboratory, RIKEN Center for Developmental Biology, Kobe, Hyogo, Japan; 2 CREST, Japan Science and Technology Agency, Kobe, Hyogo, Japan; Casey Eye Institute, United States of America

## Abstract

Establishment of apical-basal polarity is crucial for epithelial sheets that form a compartment in the body, which function to maintain the environment in the compartment. Effects of impaired polarization are easily observed in three-dimensional (3-D) culture systems rather than in two-dimensional (2-D) culture systems. Although the mechanisms for establishing the polarity are not completely understood, signals from the extracellular matrix (ECM) are considered to be essential for determining the basal side and eventually generating polarity in the epithelial cells. To elucidate the common features and differences in polarity establishment among various epithelial cells, we analyzed the formation of epithelial apical-basal polarity using three cell lines of different origin: MDCK II cells (dog renal tubules), EpH4 cells (mouse mammary gland), and R2/7 cells (human colon) expressing wild-type α-catenin (R2/7 α-Cate cells). These cells showed clear apical-basal polarity in 2-D cultures. In 3-D cultures, however, each cell line displayed different responses to the same ECM. In MDCK II cells, spheroids with a single lumen formed in both Matrigel and collagen gel. In R2/7 α-Cate cells, spheroids showed similar apical-basal polarity as that seen in MDCK II cells, but had multiple lumens. In EpH4 cells, the spheroids displayed an apical-basal polarity that was opposite to that seen in the other two cell types in both ECM gels, at least during the culture period. On the other hand, the three cell lines showed the same apical-basal polarity both in 2-D cultures and in 3-D cultures using the hanging drop method. The three lines also had similar cellular responses to ECM secreted by the cells themselves. Therefore, appropriate culture conditions should be carefully determined in advance when using various epithelial cells to analyze cell polarity or 3-D morphogenesis.

## Introduction

**Figure 1:**
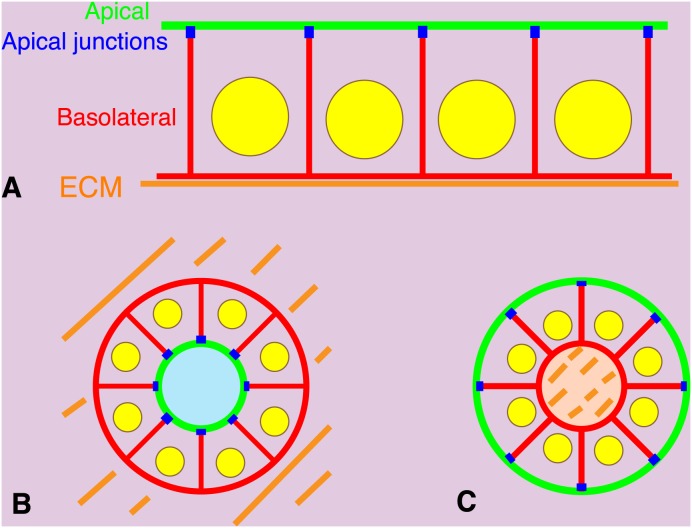
Apical-basal polarities of epithelial cells in 2-D or 3-D culture. (A) Polarized epithelial cells in a 2-D sheet. Cells are on extracellular matrix (ECM, orange) coated artificially or deposited by the cells themselves. Plasma membranes facing the ECM or adjacent cells are called basolateral membranes (red). The remaining membrane areas are called apical membranes (green). Apical junctions (blue) are formed at the border between basolateral and apical membranes. (B) Polarized epithelial cells forming a spheroid in the ECM gel. Basolateral membranes are formed on the outside surface of the spheroid facing the ECM. Apical membranes are formed inside the spheroid. (C) Polarized epithelial cells forming a spheroid in suspension culture. Concentration of the ECM deposited by the cells themselves appears higher within the spheroid. Apical membranes are formed on the outside surface of the spheroid facing the culture medium. Basolateral membranes are formed on inside the spheroid.

Epithelial sheets in multicellular organisms form physiological barriers separating the internal environment from the external environment [1]. Transport of nutrients across these sheets and directional secretion of materials from epithelial cells are required to maintain a stable internal environment. Polarization of epithelial cells is one feature essential for maintaining this environment. The epithelial plasma membrane is divided into two regions, an apical membrane facing the lumen or external environment and a basolateral membrane contacting adjacent cells and the underlying extracellular matrix (ECM). These two membrane regions have distinct functions and molecular constituents. At the border of these two regions, in the vicinity of the most apical position along the basolateral membrane, are apical junctions composed of tight and adherens junctions (Fig. 1A). Cell structures such as cilia or microvilli also show biased localization. This epithelial cell polarity is called apical-basal polarity [2]. One of apical markers is atypical protein kinase C (aPKC), consisting of PKC zeta and iota in human, which plays an essential role in cell polarity as a complex with several proteins such as Par 6. Scrib forms a complex with Discs large and Lethal giant larvae which is necessary for apical-basal polarity and is localized to the basolateral membrane [3]. ZO-1 is a scaffoliding protein localized to tight junctions in polarized epithelial cells [1].

The mechanisms underlying the establishment of apical-basal polarity are not completely understood. Effects of depletion of polarity proteins on the apical-basal polarity in epithelial sheets are often weak in two-dimensional (2-D) culture conditions on hard substrates, but are significant in three-dimensional (3-D) culture conditions in ECM gels [4]–[6], indicating that epithelial cells in 3-D culture show higher sensitivity to disturbances to the apical-basal polarity. When epithelial cells are embedded in ECM gels to form cell aggregates called spheroids, the outer surface of the spheroid facing the ECM becomes basal membranes, and apical membranes are formed on opposing inner surface, typically forming a central lumen (Fig. 1B). In contrast, when epithelial cells are cultured in suspension without touching the ECM, apical membranes are formed on the outer surface of the spheroid with basolateral membranes and cell-cell contact regions forming on the opposite side of apical membranes. Cells also secrete their own ECM, which is concentrated on the inside of the spheroid (Fig. 1C).

β1 integrin which receives and transduces signals from the ECM is deeply involved in the epithelial polarization [7], [8]. In fact, basement membranes composed of the ECM underlie all epithelial cell sheets in tissues and appear to determine the basal side of the apical-basal polarity. When digestive tracts were isolated from sea urchin embryos and cultured in sea water in the absence of ECM molecules, the outer surface of the tissue that had been the basal membrane of the epithelial cells began to generate cilia, indicating the reversal of the apical-basal polarity [9]. When spheroids of isolated thyroid cells formed by suspension culture with apical membranes on their outer surfaces were embedded inside a collagen gel, the apical-basal polarity was also reversed [10]. MDCK cells were also used to further analyze the reversal of the polarity [11].

For *in* *vitro* 3-D culture experiments, MDCK, MCF-10A [12], or Caco-2 cells [13] have often been used. Other cell types have not been examined in detail, and we do not know the common features or differences in the apical-basal polarization among the various epithelial cells. Therefore, we tested two other types of epithelial cells and MDCK II cells as a control in this study. Requirement of the ECM species in epithelial polarization and lumen formation were found to be different among the three cell types.

## Materials and Methods

### Cells and Cell culture

The following cell types were used in our experiments: MDCK II cells from dog renal tubules (provided by M. Murata, Tokyo University, Japan) [14], EpH4 cells from mouse mammary glands (provided by E. Reichman, University Children’s Hospital, Switzerland) [15] and R2/7 cells (subclones of DLD-1 cells from human colon cancer provided by F. van Roy, Ghent University, Belgium) [16] expressing wild-type mouse αE-catenin (R2/7 α-Cate) [17]. Cells were cultured in DMEM with 10% fetal bovine serum (FBS).

### 3-D Cultures

Matrigel culture: Matrigel (BD Biosciences) was added onto coverslips and allowed to set at 37°C for 15 min. Cells (5.0×10^4^ cells per well of a 24-well plate) were suspended in 2.0% Matrigel in DMEM containing 10% FBS, and layered on the top of the gel. Two or three days later, the gels containing cells were fixed and stained.

Collagen gel culture: A solution of 6×10^4^ cells in 90 µl of ice-cold DMEM containing 10% FBS and 20 mM Hepes, pH 7.4 mixed with 60 µl of ice-cold acidic bovine type-I collagen solution (IPC-50, KOKEN) was added to each Transwell filter insert fitted for a 12 well-plate (#3460, Corning). Final collagen concentration was 2 mg/ml. The plate was then incubated for 10 min at 37°C, and pre-warmed DMEM containing 10% FBS was added both on the gel (500 µl) and outer chamber (1.5 ml). The plate was placed in a CO^2^ incubator for 2–3 days [18].

Hanging drop culture: Drops of DMEM containing 10% FBS and 300 cells were placed on a square petri dish (351112, BD). Volume of each drop was 30 µl. The dish was then overturned and placed in a CO^2^ incubator for 1.5–2 d.

Lipidure-coat plates: For better live imaging, we used non-adherent Lipidure-coat 96-well U-bottom plates (A-U96, NOF) [19]. In each well, 150 µl of DMEM containing 10% FBS and 10–300 cells was added, and the plate was placed in a CO^2^ incubator for 1–2 h until most of cells settled at the bottom of the well. The plate was then set under a microscope for live imaging.

Reversal of epithelial polarity: MDCK II cell aggregates formed by hanging drop culture for 1.5–2 days were collected and seeded on Matrigel as described earlier. The cell aggregates were then further cultured for 7 days.

### Antibodies and Immunofluorescence Staining

The following primary antibodies were used: anti-ZO-1 mouse monoclonal antibody (T8-754; gifts from Sa. Tsukita, Osaka University, Japan), anti-Scrib goat polyclonal antibody (sc-11049, Santa Cruz) and anti-PKC zeta rabbit polyclonal antibody (sc-216, Santa Cruz). Alexa Fluor 488-conjugated donkey anti-mouse IgG (H+L) (A21202, Invitrogen), Alexa Fluor 555-conjugated donkey anti-goat IgG (H+L) (A21432, Invitrogen) and Dylight 649-conjugated donkey anti-rabbit IgG (H+L) (711-495-152, Jackson Immuno Research) were used as secondary antibodies. DAPI was purchased from Invitrogen. Cells cultured on coverslips were fixed with 10% ice-cold trichloroacetic acid in water for 15 min [20]. Cells cultured by other methods were fixed with 1% formaldehyde in 0.1 M Hepes buffer, pH 7.5 for 15 min at room temperature. They were rinsed twice with PBS containing 30 mM glycine (G-PBS) and then permeabilized conventionally by treating them with 0.2% Triton X-100 in G-PBS for 10 min [21]. Cells cultured on coverslips were incubated with primary and then secondary antibodies, each for 30 min. Cells cultured by the hanging drop method were collected by conventional centrifugation for cells, fixed and transferred to 0.5 ml microtubes. They were washed by centrifugation and incubated with primary and then secondary antibodies at room temperature, each for between 2 hrs to overnight. Cells were washed in a 0.5 ml microtube with G-PBS containing 0.1% Triton X-100 to prevent cells from sticking to the wall of the tube. Cells in a 0.5 ml microtube were collected using a compact personal centrifuge by ∼10 second centrifugation. For cells cultured in Matrigel, they were incubated with antibodies for 2–4 hrs. For cells cultured in collagen gels, the culture medium was removed, the gels were washed twice with DMEM without FBS, and 150 µl of collagenase type VII (C2399, Sigma-Aldrich) in DMEM (100 U/ml) was added on top of the gels in the insert. The gels were then incubated for 15 min at room temperature. Before fixation, gels were washed twice with DMEM. Duration of fixation, Triton-treatment, incubation with antibodies and washing (5 times) were 30 min, 30 min, overnight, and 30 min, respectively.

### Fluorescence Microscopy and Live Imaging

Confocal images were taken with a TCS SP8 microscope (Leica) with an HC PL APO CS2 63×/1.40 OIL and an HCX PL APO 40×/0.85 DRY lens. 3-D images reconstructed from optical sections were generated with this system. For quantitative observations, an Olympus BX51 microscope with a UPlanFL N 20×/0.50 Ph1 lens and a UPlanFL N 40×/0.75 Ph2 lens was used. For live imaging of cell aggregates in Lipidure-coat plate, an Olympus IX81 inverted microscope equipped with a sample stage for multi-point image acquisition, a CO^2^ incubator, and a cooled CCD camera (ORCA ER, 1,344 pixels ×1,024 pixels: Hamamatsu Photonics) controlled by the software package MetaMorph version 7.6.5 (Molecular Devices) was used. Time-lapse images were recorded at every 30 min for 1 day using a UPlanFL N 10×/0.30 Ph1 lens.

### Quantitation of epithelial polarity establishment

Spheroids formed under several conditions were stained for ZO-1, Scrib and PKC zeta as described earlier to identify apical areas (PKC zeta) [3], apical junctions (ZO-1) [22], and basolateral areas (Scrib) [23]. When ZO-1 was found distributed on the surface of a spheroid, typically as a network, and PKC zeta also accumulated at the surface, the spheroid was classified as “TJ outside”, meaning that apical areas are formed on the side facing outer surface of the spheroid and that basolateral areas are formed inside the spheroid. When PKC zeta accumulated to a single central position inside a spheroid, and is enclosed with a ZO-1 network indicating the existence of single lumen, the spheroid is classified as “single lumen”, meaning that basolateral areas are facing the outer surface of the spheroid and that apical areas are on the inside the spheroid. When several lumens are observed, the spheroid is classified as “multiple lumens”, meaning that epithelial polarity is similar to spheroids with a single lumen but that there is no coordinated lumen formation.

## Results

### Apical-basal polarities of epithelial cells in 2-D culture

To determine the common features and variations in the mechanism of apical-basal polarity establishment seen in many epithelial cells, focusing especially on the role of ECM, we chose three types of epithelial cell lines: MDCK II cells, EpH4 cells, and R2/7 α-Cate cells. MDCK II cells are derived from the dog kidney, and are widely used in research as a model of polarized epithelial cells [14]. EpH4 cells are derived from mouse mammary glands, and also show clear apical-basal polarity [15], [24]. R2/7 α-Cate cells are R2/7 cells, a line subcloned from DLD-1 derived from human colon cancer and lacks α-catenin expression [16], that have recovered normal cell-cell adhesion through the introduction of mouse wild-type α-catenin [17].

**Figure 2:**
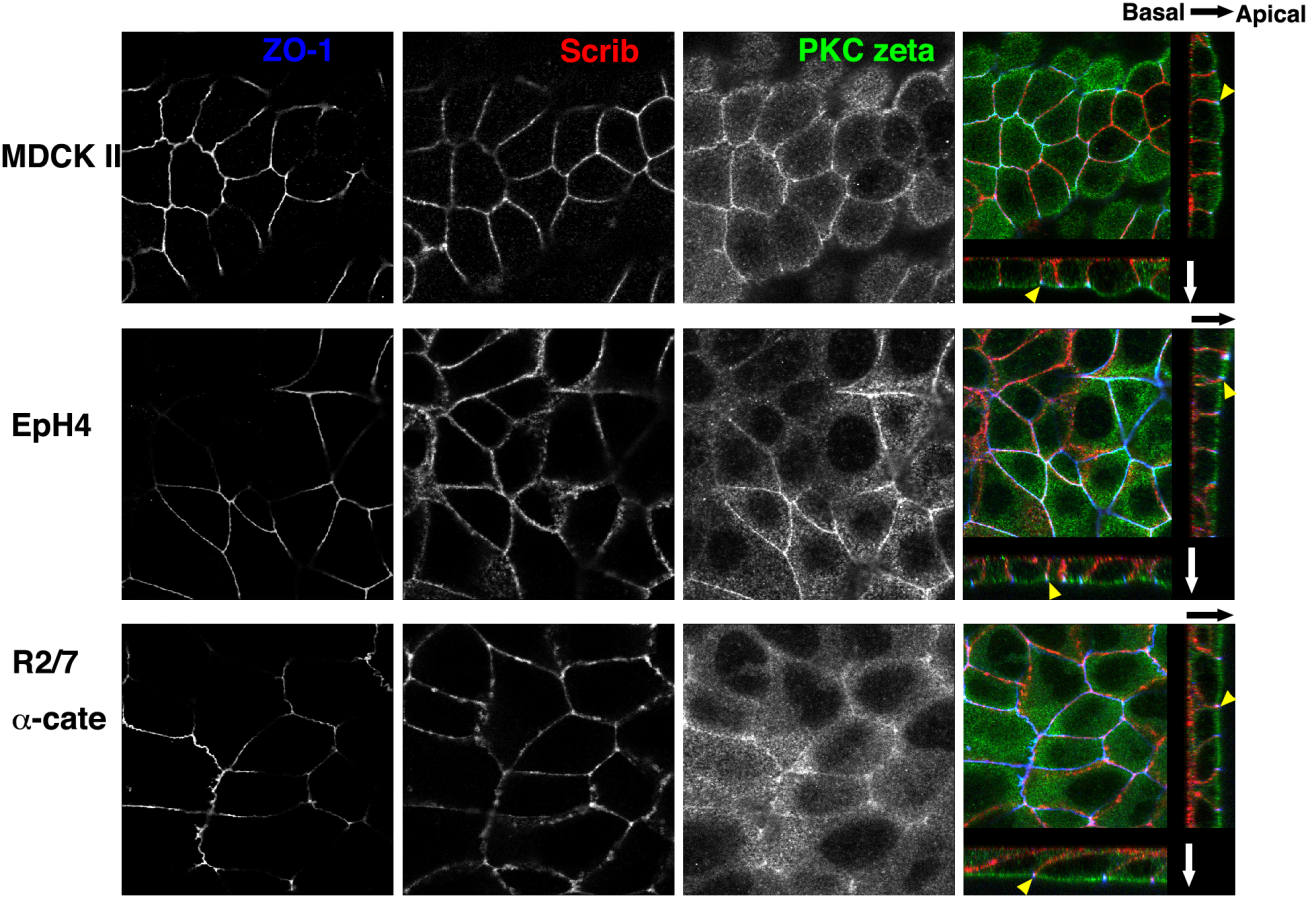
Apical-basal polarities of epithelial cells in 2-D sheets. Confluent MDCK II, EpH4, and R2/7 α-cate cells cultured on coverslips without ECM-coating were stained for ZO-1, Scrib, and PKC zeta, shown in blue, red and green, respectively in the merged images (the most right). In black-and-white images, samples were focused at the level of the apical junctions. In the merged images, series of confocal optical sections were reconstructed to show horizontal images (center) and vertical images (right and bottom). Directions of the arrows indicate from basal to apical. In all cases, PKC zeta and Scrib accumulated at apical membranes and basolateral membranes, respectively. ZO-1 was localized exclusively to the border between the two membranes, indicating the apical junction regions (yellow arrowheads). The combination of antibodies to these proteins clearly reveals similar polarization of epithelial cells of different species and origins. Bar, 10 µm.

We first confirmed that the three cell lines show typical apical-basal polarity in 2-D cultures using coverslips as a substrate (Fig. 1A). Because we did not coat the coverslips with ECM before culturing, the coverslips should have been coated with ECM secreted by the cells themselves. Confluent epithelial sheets were fixed, stained with several antibodies, and then imaged by confocal microscopy. We tried to find good markers for apical membranes, basolateral membranes, and apical junctions as the borderline of the two membranes. ZO-1, which is already widely used as a tight junction marker, clearly represented apical junctions for all three cell lines (Fig. 2, blue and yellow arrowheads in the merged panels). Scrib [25], a member of the scribble complex consisting of Scrib, lethal (2) giant larvae homologue (LGL) and discs-large homologue (DLG) localized clearly to basolateral membranes but not to apical membranes in all three cell lines (Fig. 2, red in the merged panels). aPKC, a member of the Par polarity complex consisting of Par3 and Par6, is known to play an essential role in cell polarity [25], and one aPKC member, PKC zeta, was found to localize preferentially to apical membranes in all cases (Fig. 2, green in the merged panels). Merged images at the most right are serial optical sections reconstructed to show horizontal images (center) and vertical images (right and bottom). ECM-coated substrates appeared to determine the position of the basal membrane, which also eventually orients the apical-basal polarity in these cell lines. Scrib and aPKC are important for apical-basal polarity establishment and are distributed ubiquitously, and tight junctions are common to all polarized epithelial cells. Therefore, apical-basal polarity of a range of epithelial cells could be revealed by examining the localization of the combination of these proteins.

### Apical-basal polarities of epithelial cells in spheroids formed in 3-D culture

**Figure 3:**
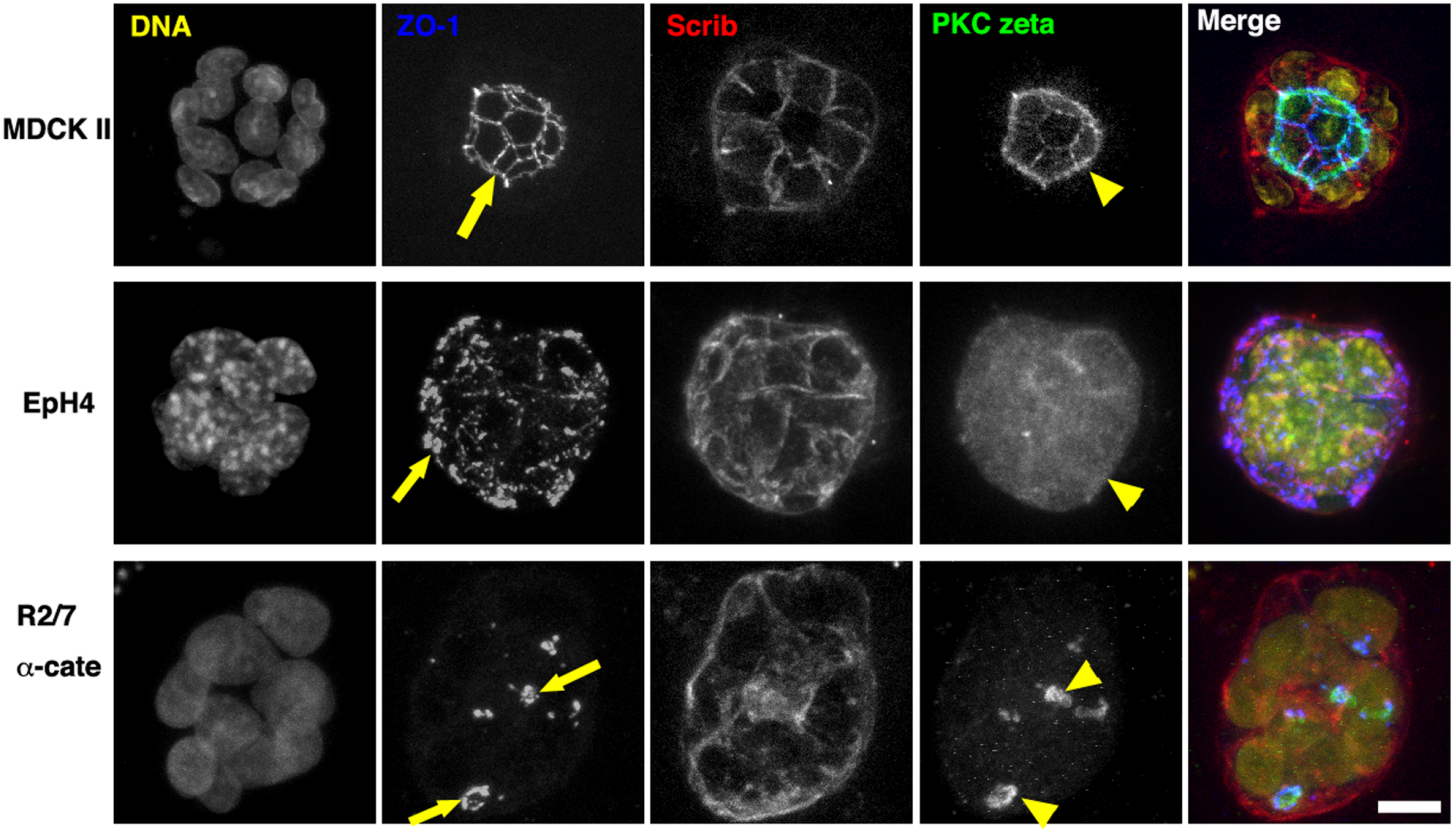
Apical-basal polarity and lumen formation of epithelial spheroids in Matrigel. Spheroids of epithelial cells after culturing for 2 days (EpH4 cells) or 3 days (MDCK II and R2/7 α-Cate cells) in Matrigel. They were stained for DNA (yellow), ZO-1 (blue), Scrib (red), and PKC zeta (green). Series of confocal optical sections were obtained and projected on a single plane. In MDCK II cells, single lumens lined with PKC zeta (yellow arrowhead) and enclosed by a ZO-1 network (yellow arrow) were observed. EpH4 cells typically had no lumen and ZO-1 was distributed on the surface of the spheroid (yellow arrow). R2/7 α-Cate cells had several small lumens inside the spheroid (yellow arrows and arrowheads). Bar, 10 µm.

Compared with the 2-D culture on a very hard substrate, the 3-D culture in an ECM gel is closer to physiological conditions (Fig. 1B). We first embedded the cells in Matrigel, which contains laminin, entactin, and collagen IV, and cultured them for 2–3 days. In all cases, we observed spheroid formation. The ECM gels containing spheroids were then fixed, and stained with several antibodies. Series of confocal optical sections were obtained and projected on a single plane (Fig. 3). In MDCK II cells cultured for 3 days, the spheroids had a single central lumen lined with PKC zeta (yellow arrowhead), which was enclosed by a tight junction network (yellow arrow) (see also Fig. 1B). Scrib was localized both to lateral membranes and to basal membranes facing the Matrigel. The EpH4 cells typically had no lumen, and ZO-1 was distributed on the surface of the spheroid (yellow arrow), indicating that the polarity is reversed (see also Fig. 1C). They were cultured for 2 days because cell death was observed during prolonged culture in Matrigel. The R2/7 α-Cate cells showed similar apical-basal polarity to that seen in MDCK II cells, but, the spheroid had several small lumens (yellow arrows and arrowheads). Thus, these three cell lines responded differently to the same ECM, Matrigel, in terms of apical-basal polarity and lumen formation, even though they showed similar apical-basal polarities in 2-D cultures.

**Figure 4:**
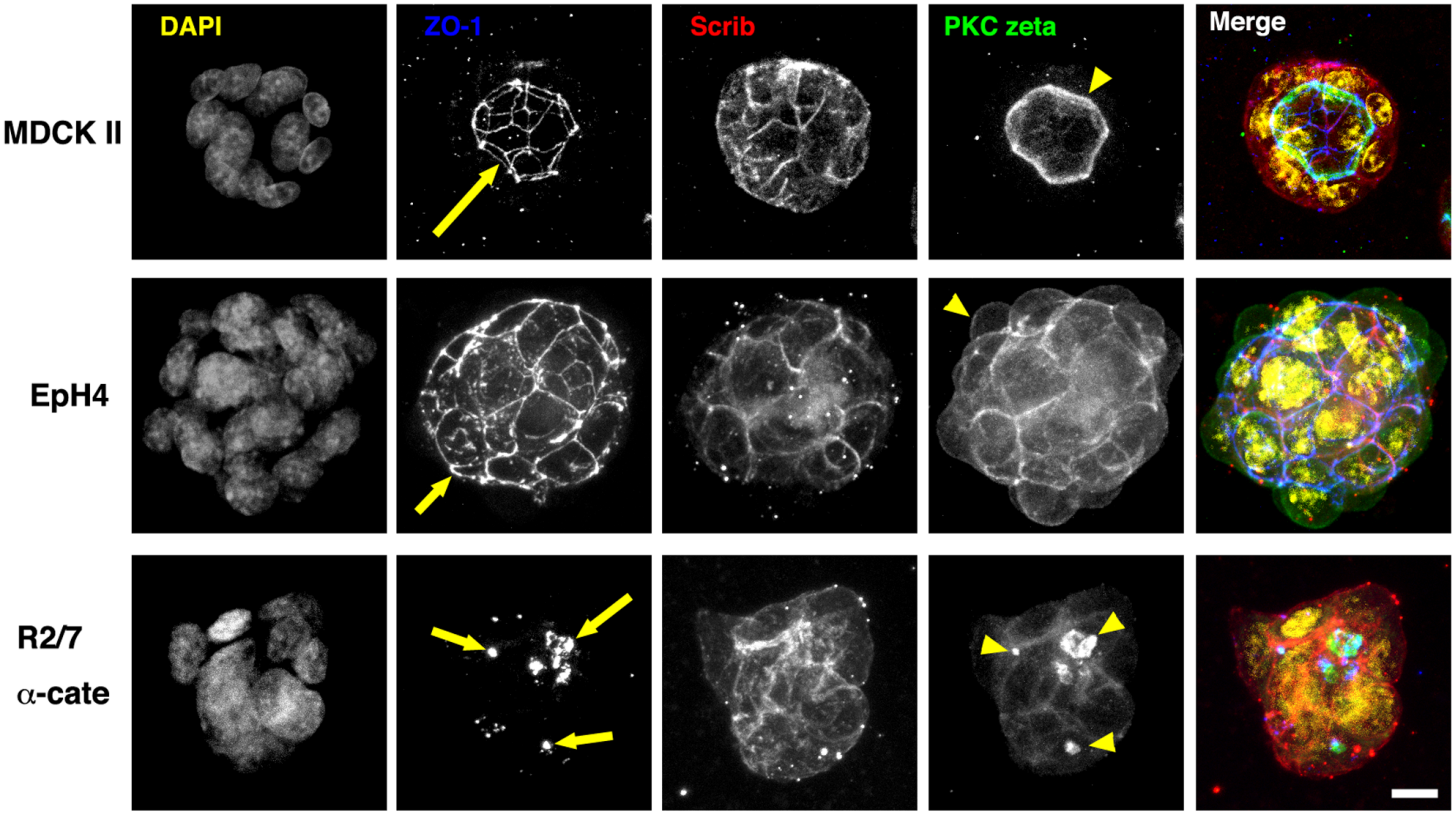
Apical-basal polarity and lumen formation of epithelial spheroids in collagen gels. Spheroids of MDCK II, EpH4, and R2/7 α-cate cells after 3-day culture in type I collagen gels. Cells were stained for DNA (yellow), ZO-1 (blue), Scrib (red) and PKC zeta (green). Series of confocal optical sections were obtained and projected onto a single plane. In MDCK II cells, single lumens lined with PKC zeta (yellow arrowhead) and enclosed by ZO-1 network (yellow arrow) were observed. EpH4 cells had no lumen, and the ZO-1 network (yellow arrow) was distributed on the surface of the spheroid. PKC zeta also accumulated at the surface of the spheroid (yellow arrowhead). R2/7 α-Cate cells had several small lumens inside the spheroid (yellow arrows and arrowheads). Bar, 10 µm.

We then tried culturing cells for 3 days in collagen type I gels (Fig. 4). The results of 3-D culture in collagen gels were similar to those in 3-D culture in Matrigels. For MDCK II cells, the constituents in Matrigel (laminin, entactin, collagen IV, etc.) and collagen type I gels appeared to function as the same signal that determines the apical-basal polarity. On the other hand, EpH4 cells showed quite different responses to the ECM gels. None of the constituents in the Matrigel or the collagen gel appeared to function in determining the basal membranes in EpH4 cells. The cell responses of R2/7 α-Cate cells to the ECM gels were similar to that seen in MDCK II cells, although the R2/7 α-Cate cells lacked the coordination to form a central lumen.

**Figure 5:**
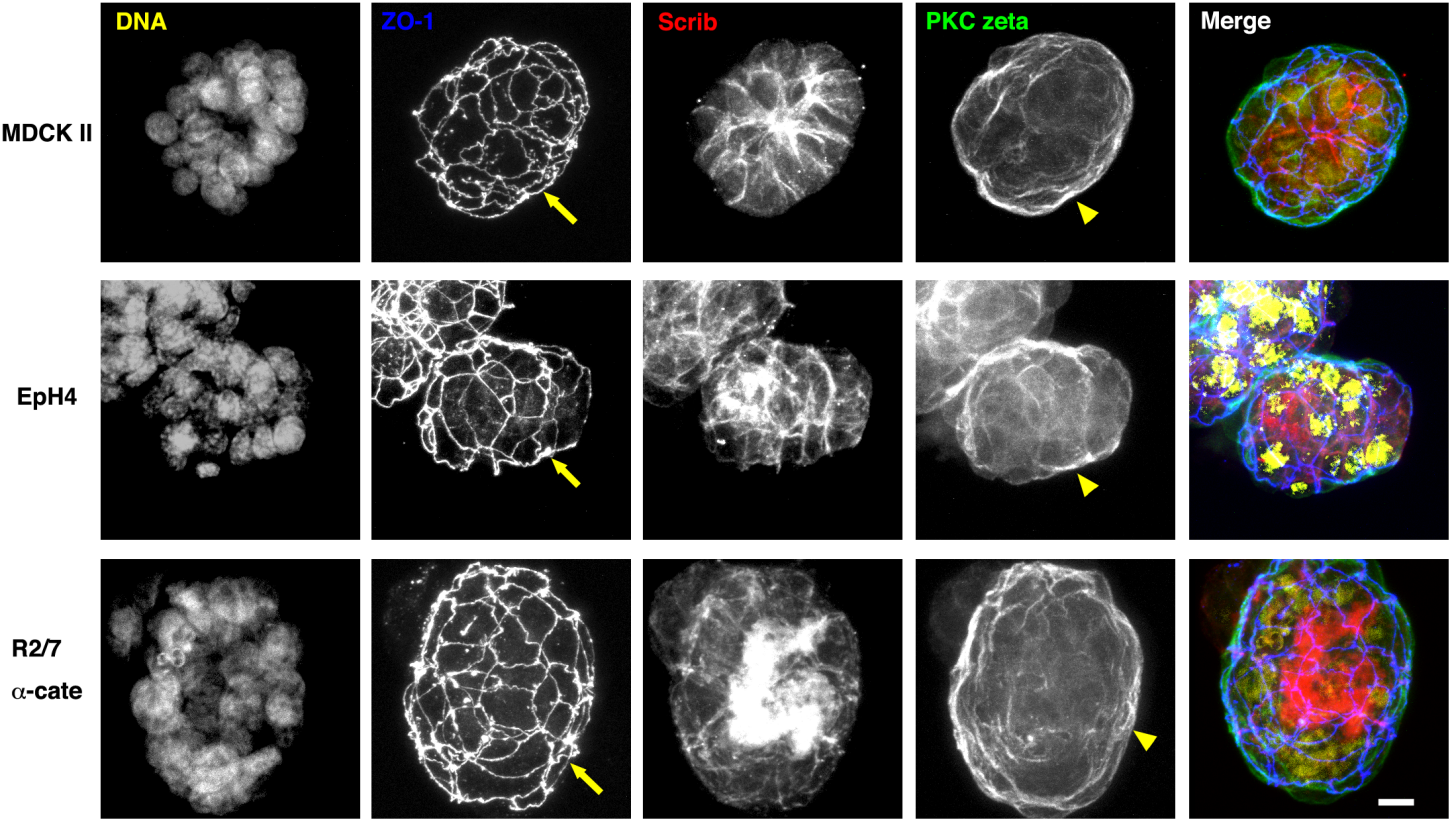
Apical-basal polarity formation of epithelial spheroids in hanging drops. Spheroids of MDCK II, EpH4 and R2/7 α-Cate cells after 1.5–2 day culture in hanging drops. In this case, the ECM deposited by those cells is considered to be concentrated within the spheroids. Spheroids were stained for DNA (yellow), ZO-1 (blue), Scrib (red) and PKC zeta (green). Series of confocal optical sections were obtained and projected onto a single plane. In MDCK II cells PKC zeta accumulated on the surface of the spheroid (yellow arrowhead). A ZO-1 network was also found at the surface (yellow arrow). The epithelial cell polarity is opposite to that of cells in the ECM gels. EpH4 and R2/7 α-Cate cells showed similar protein distributions, and also showed similar polarities. Bar, 10 µm.

To observe 3-D morphogenesis of epithelial cells dependent on the ECM they secrete themselves, we also tried the hanging drop culture method (Fig. 1C). In a hanging drop of culture medium, cells gather at the interface between the bottom of the drop and the surrounding air due to forces of gravity and then form an aggregate. The concentration of ECM secreted by the cells was considered to be high in the center of the aggregate and low in the culture medium. Cells were cultured for 2 days, and the resulting spheroids were collected, fixed, stained with several antibodies, and imaged by confocal microscopy (Fig. 5). As expected, the outer surfaces of the spheroids were judged to be apical membrane based on PKC zeta accumulation and the existence of tight junction networks in all cases, indicating that the direction of apical-basal polarity is opposite to that seen in spheroids formed when cultured in ECM gels. There was also no obvious interior space in the spheroids. EpH4 cells showed a clear apical-basal polarity similar to other two cell types with ECM found in the interior of the spheroid, indicating that ECM molecules secreted specifically by EpH4 cells, but not contained in Matrigel or collagen gel, are responsible for the determination of apical-basal polarity in EpH4 cells.

**Figure 6:**
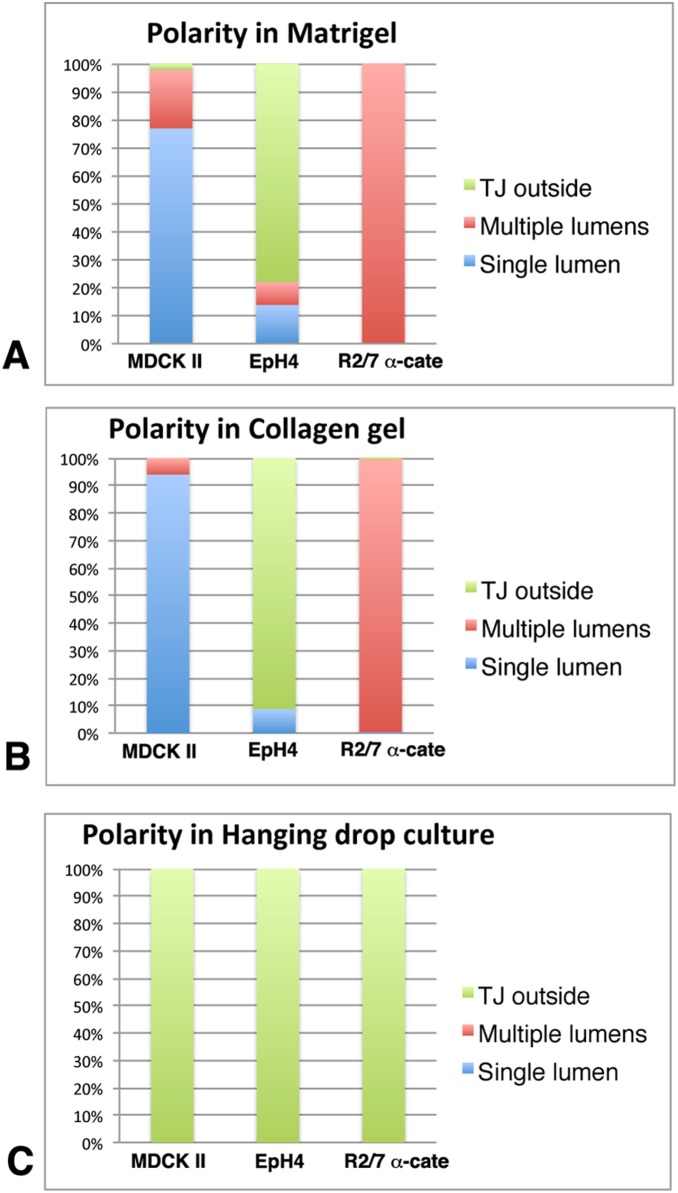
Quantitation of apical-basal polarity and lumen formation in epithelial spheroids. Polarity and lumen formation of spheroids cultured under different conditions shown in Figs. 3–5 was quantified by microscopic observation. “TJ outside” means a spheroid with apical membranes connected with tight junctions facing the outside (see Fig. 1C). “Single lumen” means a spheroid with basolateral membrane facing the outside, apical membrane inside, and only one lumen inside (see Fig. 1B). “Multiple lumens” means a spheroid with basolateral membrane facing the outside, apical membrane inside, and several lumens inside. (A) Cells were cultured in Matrigel for 3 days with the exception of Eph4 cells which were cultured for 2 days. While MDCK II and R2/7 α-Cate spheroids show the same polarity (apical inside), MDCK II spheroids typically have a single lumen whereas R2/7 α-Cate spheroids have multiple lumens. Most of the EpH4 spheroids showed opposite polarity. n = 442, 409 and 570 for MDCK II, EpH4 and R2/7 α-Cate spheroids, respectively, from 3–4 independent experiments. (B) Cells were cultured in collagen gel for 3 days. The results were similar to those cultured in Matrigel. n = 339, 280 and 425 for MDCK II, EpH4 and R2/7 α-Cate spheroids, respectively, from independent 3–4 experiments. (C) Cells were cultured in hanging drops for 1.5–2 days. Spheroids of all cell types showed the same polarity (apical outside). n = 387, 153 and 378 for MDCK II, EpH4 and R2/7 α-Cate spheroids, respectively, from independent 3 experiments.

We further quantitated the establishment of apical-basal polarity and the formation of lumens in those spheroids (Fig. 6). Spheroids were classified as “TJ outside” when PKC zeta accumulation and a tight junction network, but no Scrib, were found at the outer surface of a spheroid, meaning that the apical membranes are located on the outer surface of the spheroid (see also Fig. 1C). Spheroids with PKC zeta accumulation and a tight junction network observed in the interior of a spheroid and with Scrib distributed to the outer surface were classified as “single lumen” or “multiple lumens” according to the number of lumens they had; for both categories, the apical membranes were located in the interior of the spheroid (see also Fig. 1B). Most of the MDCK II spheroids formed both in Matrigel and collagen gel showed a single lumen (Fig. 6A and B). Under the same conditions, however, all spheroids of R2/7 α-Cate cells had multiple lumens. EpH4 spheroids typically showed apical-basal polarity which was opposite of MDCK II and R2/7 α-Cate cells. In the hanging drop cultures, spheroids of all cell lines showed a “TJ outside” phenotype (Fig. 6C).

### Reversal of the apical-basal polarity

**Figure 7:**
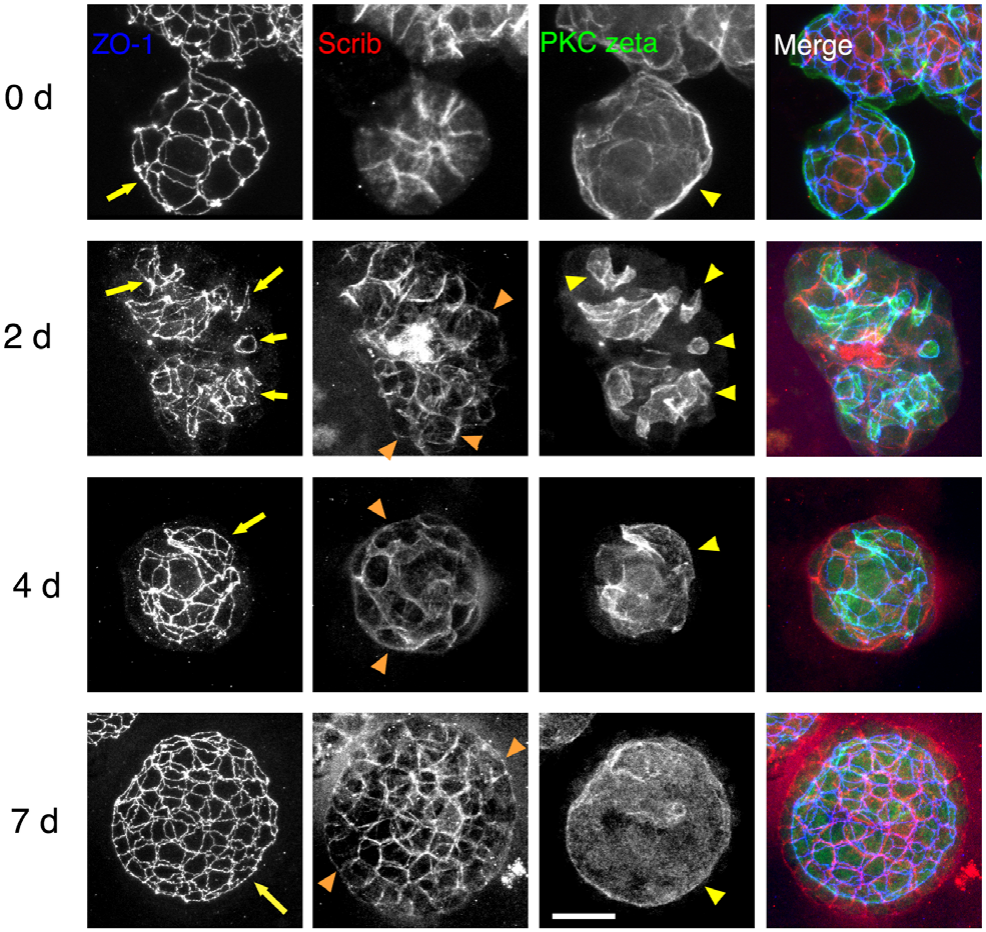
Reversal of apical-basal polarity of MDCK II spheroids. MDCK II spheroids cultured in hanging drops for 1.5–2 days were collected and further cultured in Matrigel. Spheroids were stained for ZO-1 (blue), Scrib (red), and PKC zeta (green). Series of confocal optical sections were obtained and projected onto a single plane. On day 0 just before starting culture in Matrigel, spheroids showed polarity, with the apical membrane facing the outside. Spheroids cultured for 2 days in Matrigel had several lumens inside associated with PKC zeta (yellow arrowheads) and ZO-1 accumulation (yellow arrows), indicating that the reversal of epithelial apical-basal polarity is taking place. Scrib began to be redistributed to the outside surface of the spheroids (orange arrowheads). Spheroids cultured for 4 days in Matrigel showed fusion of multiple lumens to form a single lumen (yellow arrow and yellow arrowhead). Spheroids cultured for 7 days in Matrigel showed an expanded single lumen associated with PKC zeta (yellow arrowhead) and ZO-1 network (yellow arrow). The epithelial apical-basal polarity has been completely reversed. Bar, 20 µm.

Although several suspension culture methods can be used to form spheroids with their outer surfaces facing the culture medium [26], the sizes of the spheroid cannot be controlled. But we were able to obtain spheroids of similar sizes by changing the number of cells contained in the hanging drop, which were then easy to collect and use in other experiments. Using this to our advantage, we collected MDCK II spheroids formed in hanging drops for 1.5–2 days and embedded them into Matrigel. It is known that the epithelial apical-basal polarity can be reversed experimentally by changing the environment; when spheroids grown in suspension culture with apical membrane at their outer surfaces are transferred into ECM gels, the outer surfaces of the spheroids eventually become the basal membrane, and they have lumens with apical membranes inside [9]–[11]. As expected, the polarity of spheroids were reversed after transferring them into Matrigel (Fig. 7). Two days after the transfer, several lumens were observed in the interior of spheroids, and 4 days after the transfer, they appeared to fuse and form an almost single lumen. At 7 days after the transfer, cells formed a monolayer and a single large lumen was observed. Thus, a further detailed analysis would be possible using this experimental system.

### Time-lapse observation of spheroid formation

**Figure 8:**
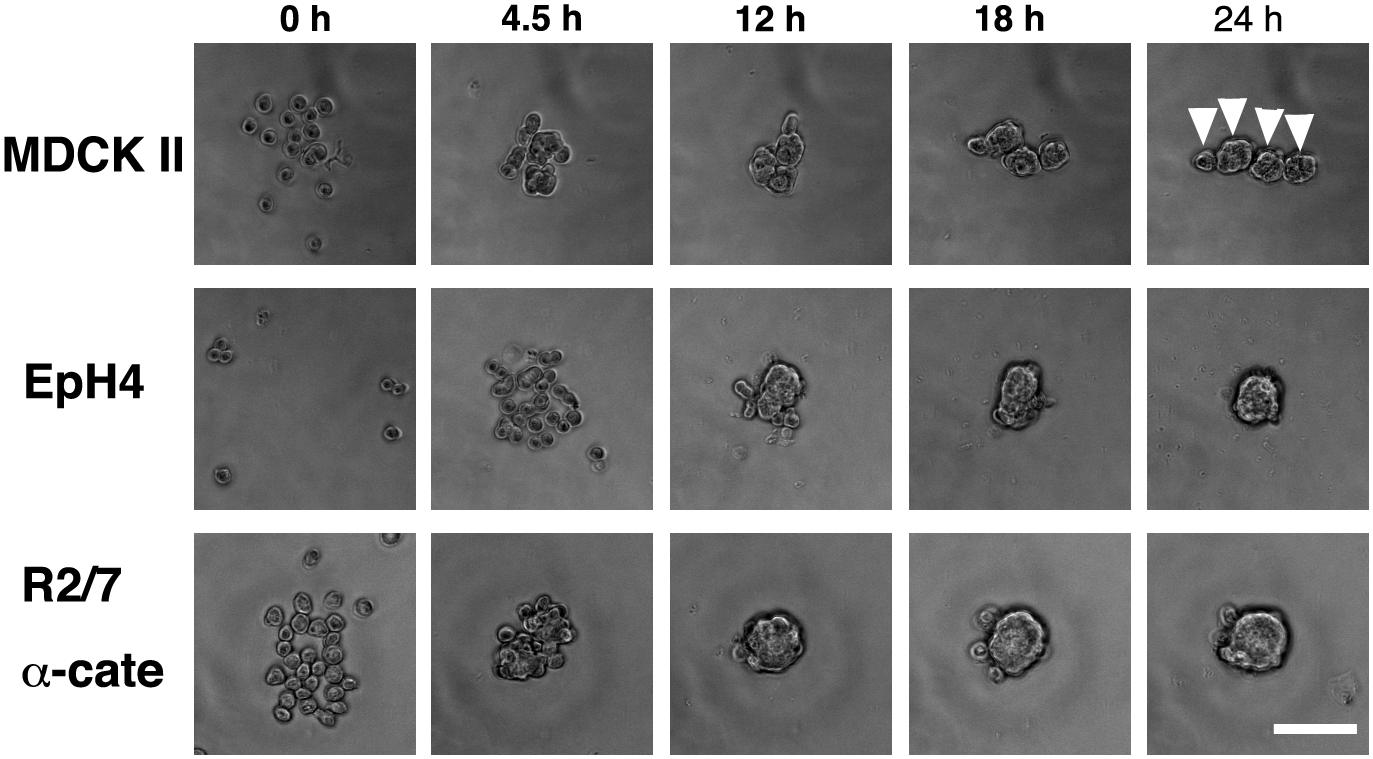
Spheroid formation in Lipidure-coated plates. Cells were cultured in non-adherent Lipidure-coated 96-well U-bottom plates for time-lapse recording over 24 hrs. MDCK II cells attached together quickly to form several spheroids, each consisting of a small number of cells. The spheroids themselves did not fuse together, remaining separate from each other (white arrowheads). EpH4 cells did not attach together quickly, and tended to form a single spheroid. R2/7 α-Cate cells attached together quickly and tended to form a single spheroid. Bar, 100 µm.

We attempted to record the morphogenetic processes of spheroid formation using live-imaging. Because establishment of similar apical-basal polarity in all three cell lines was possible using the hanging drop method, we first chose this method for live imaging. Unfortunately, spheroids in hanging drops turned out to be unsuitable for imaging as the distance between the hanging drops on the lid of a culture plate and the objective lens of an inverted microscope was too long to obtain images at appropriate magnification. The drops themselves also acted as a lens and optically distorted the images of spheroids. Therefore, we searched for U-bottomed 96 well-plates with walls that did not allow cell attachment, and found Lipidure-coated plates to be suitable for our purposes [19]. Similar to hanging drops, cells aggregate at the bottom of each well due to the gravitational forces. The Lipidure-coat prevents cells from adhering to the walls of the plates, yet allows cells to migrate along the walls to some degree. Although a U-shaped bottom was not optically ideal for imaging, the contour of each spheroid was clearly discernible. Protein distributions in the spheroids cultured in these plates were quite similar to those cultured by the hanging drop method. Using these plates, we could record the formation of up to 96 spheroids at the same time using an inverted microscope equipped with a sample stage for multi-point image acquisition and a CO^2^ incubator. Spheroids formed faster in Lipidure-coated plates than in the hanging drop probably because cells could not migrate efficiently in the drop to form a single aggregate at the interface between the hanging drop and air. When the number of cells at the beginning of culture was relatively small (20–30), EpH4 and R2/7 α-cate cells formed a single, almost round spheroid in each well within 24 hrs. In contrast, MDCK II cells formed several small spheroids in each well (Fig. 8). But, because there was no cell-cell adhesion at the beginning of the culture, these MDCK II cell spheroids did not indicate the existence of several small cell aggregates that could not be dissociated during experimental procedure (Fig. 8, 0 h, movie S1). The small spheroids were formed at 4–6 hrs after the beginning of culture and did not fuse together as time progressed (movie 1). EpH4 cells were slow to gather to form a cell aggregate compared to the other two cell types. Approximately 8 hrs after the beginning of culture, EpH4 cells appeared to form a cell aggregate that was plastic and could continue to integrate surrounding cells to into the aggregate (movie S2). R2/7 α-cate cells also formed a cell aggregate at 2–6 hrs after the beginning of culture. The aggregate, however, appeared plastic and deformable, and formed a single round spheroid (movie S3).

## Discussion

In this study, we tried to determine both the common features and the variations in the mechanisms of epithelial polarity establishment seen in many epithelial cells, focusing especially on the role of ECM. We used three epithelial cell lines with different origins. MDCK II, EpH4 and R2/7 α-Cate cells showed very clear apical-basal polarity in 2-D culture. But in 3-D culture, each cell type showed different responses to the same commercially available ECM. In MDCK II cells, spheroids with a single central apical lumen were formed both in Matrigel and in collagen gel. In R2/7 α-Cate cells, the spheroids that formed displayed apical-basal polarity that was similar to that seen in MDCK II cells, but they had multiple lumens. In EpH4 cells, the spheroids had apical-basal polarity that was opposite to that seen in the other two cell types in both Matrigel and collagen gel, at least during the culture period. On the other hand, all cell lines showed the same apical-basal polarization both in the 2-D cultures and the 3-D cultures using the hanging drop method. The three cell lines also displayed similar cellular responses to ECM the cells secreted themselves. Thus, it is likely that each cell line shows different expression profiles of integrin species [27]. EpH4 cells, for example, may not express sufficient amounts of the type of integrins that can receive signals from Matrigel or collagen type I. Although MDCK cells are widely known to respond both to Matrigel and to collagen gel, other epithelial cell lines do not necessarily respond to those commercial gels. Because the cells in the 3-D culture using the hanging drop method can receive signals from the ECM they secreted themselves, this system could be applied to a variety of epithelial cells for analyzing 3-D morphogenesis.

The origin of the cell line may also affect the phenotype of the resulting spheroids in different ECM gels. R2/7 α-Cate cells cultured in the ECM gels formed spheroids with multiple lumens. Although the presence of multiple lumens in R2/7 α-Cate cell spheroids might be caused by defects in the mechanism coordinating the fusion of multiple lumens into a common large lumen, the origin of the cell line may also be a factor. MDCK II cells are derived from renal tubules [14], which physiologically are surrounded by ECM on the outside and have a lumen on the inside. On the other hand, R2/7 cells are derived from the epithelium of the colon [16]. In the villi of the colon, epithelial cells are mostly surrounded by the extracellular space within the digestive tract and ECM is found on the inside. Thus, it is possible that for R2/7 α-Cate cells, the 3-D culture using the hanging drop method is closer to physiological conditions than those using ECM gels.

Using Lipidure-coated plates, we were able to obtain time-lapse images of spheroid formation. The combination of Lipidure-coated 96-well U-bottom plates with an inverted microscope equipped with a sample stage for multi-point image acquisition and a CO^2^ incubator will be useful for high-throughput analysis or screening using spheroid formation. While the quality of acquired images was sufficient for grasping the contour of the spheroids, it was not adequate for obtaining precise 3-D fluorescence images (data not shown).

This study revealed that not all epithelial cell lines behave similarly to MDCK cells in a 3-D culture system. Although MDCK cells are a very good model for analyzing epithelial polarity or morphogenesis [2], [28], [29], appropriate culture conditions should be carefully determined in advance if use of other epithelial cells is required.

## Supporting Information

Movie S1 **3-D morphogenesis of MDCK II cells in Lipidure-coated wells for 24** **hrs.** The timescale at the upper-right corner indicates the hours and minutes after the beginning of imaging. MDCK II cells attached together quickly to form several spheroids, each consisting of a small number of cells. These spheroids did not fuse together and remaining separate from each other.
(MOV)

Movie S2 **3-D morphogenesis of EpH4 cells in Lipidure-coated wells for 24** **hrs.** The timescale at the upper-right corner indicates the hours and minutes after the beginning of imaging. EpH4 cells did not attach together quickly, and tended to form a single spheroid.
(MOV)

Movie S3 **3-D morphogenesis of R2/7 α-Cate cells in Lipidure-coated wells for 24** **hrs.** The timescale at the upper-right corner indicates the hours and minutes after the beginning of imaging. R2/7 α-Cate cells attached together quickly and tended to form a single spheroid.
(MOV)
